# Gonadotropin-Releasing Hormone for Preservation of Ovarian Function during Chemotherapy in Lymphoma Patients of Reproductive Age: A Summary Based on 434 Patients

**DOI:** 10.1371/journal.pone.0080444

**Published:** 2013-11-28

**Authors:** Yaoyao Zhang, Zhun Xiao, Yan Wang, Shan Luo, Xiaohong Li, Shangwei Li

**Affiliations:** Reproductive Medical Center, Department of Obstetrics and Gynecology, West China Second University Hospital, Sichuan University, Chengdu, Sichuan, P. R. China; Baylor College of Medicine, United States of America

## Abstract

**Background:**

Gonadotropin-releasing hormone agonists (GnRHa) might play a role in preserving ovarian function in lymphoma patients by inhibiting chemotherapy-induced ovarian follicular damage. However, studies of its clinical efficacy have reported conflicting results.

**Method:**

We conducted a meta-analysis to determine the effect of the preservation of ovarian function by administering GnRHa in young patients with lymphoma undergoing chemotherapy. Seven studies were identified that met inclusion criteria and comprised 434 patients assigned to GnRHa combined chemotherapy or chemotherapy alone.

**Results:**

The incidence of women with premature ovarian failure (POF) demonstrated a statistically significant difference in favor of the use of GnRHa (OR=0.32, 95% CI 0.13-0.77). In addition, the final level of FSH in the GnRH group was significantly lower than control group. (MD= -11.73, 95% CI,-22.25- -1.20), and the final level of AMH in the GnRH group was significantly higher than control group (MD=0.80; 95% CI, 0.61–0.98). However, there was no statistically significant difference between treatment and the control groups in the incidence of a spontaneous pregnancy (OR=1.11; 95% CI, 0.55–2.26).

**Conclusion:**

This meta-analysis suggests that GnRHa may be effective in protecting ovarian function during chemotherapy in lymphoma patients. More well-designed prospective studies are needed to carry out for further understanding of this topic.

## Introduction

Over the last few decades, the number of long-term survivors with hematologic malignancies has dramatically increased. The most common significant long-term toxicity of chemotherapy in women is premature ovarian failure. Many hematologic malignancy survivors will eventually become interested in childbearing. Therefore, it is important to maximize their chances for success [1]. 

Different approaches have been developed to preserve fertility in women exposed to chemotherapy, including gametes and ovarian tissue cryopreservation[2]. However, for ovarian tissue cryopreservation, the risk of transmitting malignant cells via ovarian transplantation may be relatively high for the blood-borne cancers such as leukemia and lymphoma[3]. The most established strategy in female infertility is the cryopreservation of embryos after in vitro fertilization. However, ovarian stimulation protocol for in vitro fertilization may require up to several weeks[4]. Therefore, this procedure may not be an option for women with highly aggressive lymphoma that require immediate cytotoxic treatment[5]. 

Another option for protecting female reproductive function and for preventing ovarian damage is the administration of GnRHa during chemotherapy. The mechanisms of action by means of which of GnRH analogues preserve ovarian function are not fully understood but may include the interruption of FSH secretion, a decrease in utero-ovarian perfusion, the activation of GnRH receptors, the up-regulation of intra-gonadal anti-apoptotic molecules such as sphingosine-I-phosphate, or the protection of undifferentiated germ-line stem cells[6]. In the past few decades, fierce debates on whether GnRHa could preserve ovarian function during chemotherapy have been raised. Several clinical studies have evaluated its effect in lymphoma patients, but the results vary significantly[7-13]. The influence of GnRHa given during chemotherapy on ovarian function in is still uncertain based on these conﬂicting results. In this context, we present a concentrated systematic review and meta-analysis to summarize the available published studies regarding whether GnRHa administration before and during combination chemotherapy for lymphoma patients could preserve post-treatment ovarian function.

## Materials and Methods

### Literature Search

We conducted a search of the ClinicalTrials.gov, Cochrane Database of Clinical Trials, MEDLINE, and EMBASE with no language restrictions for relevant studies. The search terms used to identify potentially eligible studies from each data source were: “gonadotropin releasing hormone”, “GnRH”, “luteinizing-hormone releasing hormone”, “LHRH”,” “chemotherapy”, “gonadotoxicity”, “premature ovarian failure”, “menopause, premature”, “fertility”, “fertility preservation”. The last updated search was performed in May 2013. The search strategy was developed by database specialty personnel not associated with the study. Reference lists from pertinent reviews and retrieved articles were also checked to identify additional studies. In addition, we attempted to find data from poster presentations and by consulting several experts in the field. 

### Study Selection

Criteria for inclusion in the study were established before the literature search. Inclusion was limited to studies that (1)should be published studies, (2)patients had been treated with GnRH agonists concurrently with chemotherapy (GnRH group) compared to patients treated with chemotherapy alone (control group), (3)enroll study participants who were female adult cancer patients with normal menstruation before chemotherapy. Two reviewers (Z.Y.Y and W.Y.), who worked independently, used these criteria to review each article identified.

A study was excluded if: (1) The research combined treatment with GnRH antagonist and agonist; (2) the report was repetitive or some of the patients included in two studies were identical (only the most recent article was included).

### Data Collection

The two reviewers applied the eligibility criteria and assessed study quality independently. Inconsistencies between reviewers' data were resolved through discussion until a consensus was reached. The quality of case-control study was assessed using NEWCASTLE-OTTAWA QUALITY ASSESSMENT SCALE (NOS), and the two reviewers scored stars independently. The extracted data included characteristics of the study, patient populations, interventions, and outcomes. The primary outcome was the rate of POF incidences after cessation of treatment. POF is defined by the investigators in each study; secondary outcome was spontaneous pregnancy during the follow-up period after cessation of treatment and final serum FSH and AMH level. 

### Statistical Analysis

Meta-analysis was performed according to recommendations from The Cochrane Collaboration and the quality of reporting of meta-analyses guidelines[14]. The effect measures estimated were odds risk (OR) for dichotomous data and mean difference(MD) for continuous data, both reported with 95% confidence interval (CI). The proportion of heterogeneity was evaluated by Q test and I^2^ index values and reported for each outcome as P value and percentage, respectively. If I^2^ ≤50%, the variation of the studies was considered to be homogenous, the fixed effect model was adopted. If I^2^ >50%, there was significant heterogeneity between studies, the random effects model was adopted. All P values are 2-tailed, α < 0.05 was considered statistically significant (p <0.05). Analysis was performed using the statistical software Intercooled Stata version 11.0 (Stata Corp, College Station, Texas) and Review Manager version 5.2 (The Cochrane Collaboration, Oxford, United Kingdom).

## Results

### Results of the Searches

**Figure 1:**
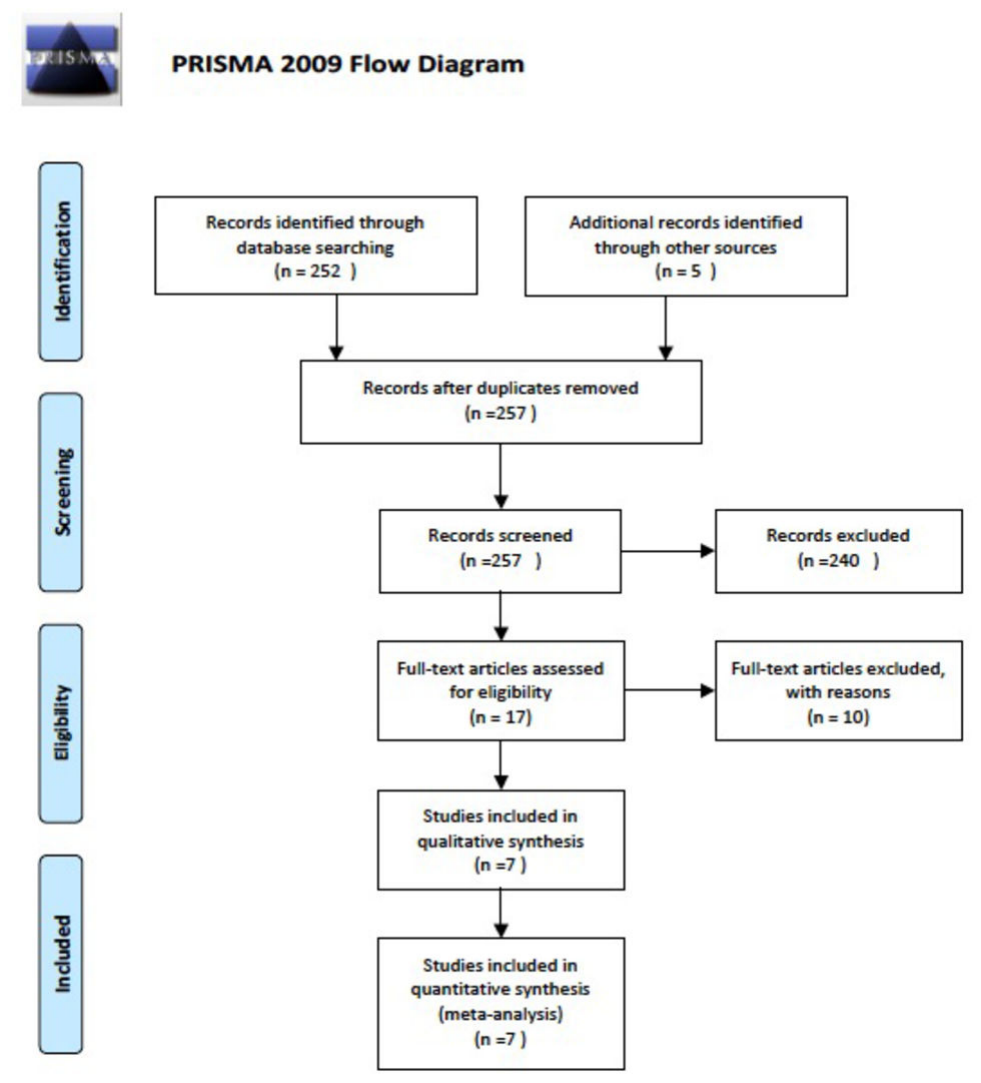
Flow diagram for selection of the studies.

The literature search yielded 257 citations. Of these, 240 were excluded after reading the title and the abstract. Full text versions of 17 articles were obtained. After full-text review, 10 studies were excluded due to differing grouping condition or repetitive data. Therefore, of these 17 articles, 7 were included in the completed review (Figure 1, Table 1)[7-13]. These studies represented a total of 434 patients with GnRHa combined chemotherapy or chemotherapy alone. Three studies were prospectively randomized studies including 130 patients (67 in the study group and 63 in the control group)[7,12,13]. Four studies were case series with control including 304 patients (177 in the study group and 127 in the control group)[8-11]. Characteristics of the included studies are listed in Table 1. 

**Table 1:** Characteristics of included studies.

Study	Method	Participants	Intervention	Outcomes	NOS stars
Demeestere et al., Hodgkin and non-Hodgkin lymphoma	multicenter, randomized, prospective trial	GnRH: 45;Control: 39	Chemotherapy: three to eight cycles of chemotherapy or high-dose therapy with autologous stem-cell transplantation as the first-line consolidative treatment;GnRH: triptorelin 11.25 mg every 12 weeks until the end of chemotherapy	Ovarian function recovery rate（FSH level≤10 IU/L), AMH level and the FSH values.	/
Giuseppe et al., Hodgkin disease	Randomized controlled trial	GnRH: 14;Control: 15	Chemotherapy: Up to 6 cycles ABVD alternating with C(M)OPP or C(M)OPP alternating with ABV. Additional DHAP in cases of incomplete remission;GnRH: Triptorelin (3.25 mg)/month or depot triptorelin (11.25 mg)/3 months for duration of chemotherapy	Incidence of spontaneous menstruation,Post-CHT markers of ovarian reserve (FSH, LH, inhibin B, AMH, AFC)	/
Waxman et al. advanced Hodgkin disease	Randomized controlled trial	GnRH: 8;Control: 10	Chemotherapy: Up to six cycles of MVPP;GnRH: Buserelin (200 mg) thrice daily intranasally for duration of chemotherapy	Incidence of spontaneous menstruation,Incidence of spontaneous pregnancy	/
Blumenfeld et al. Hodgkin lymphoma	Case–control study	GnRH: 65;Control: 46	Chemotherapy: BEACOPP , ABVD , or MOPP/ABV(D);GnRH: monthly injection administered before starting chemotherapy until its conclusion, up to a maximum of 6 months	Incidence of spontaneous menstruation,Incidence of spontaneous pregnancy,primordial follicle count on both ovaries, FSH,LH levels.	6
Huser et al. Hodgkin lymphoma	Case–control study	GnRH: 72;Control: 45	Chemotherapy: ABVD, Combination of ABVD BEACOPP regimens,BEACOPP regimen;GnRH: Triptorelin (3 mg)/a month until the end of chemotherapy	Incidence of spontaneous menstruation, endometrial thickness and primordial follicle count on both ovaries, FSH,LH levels.	5
Nitzschke et al. Hodgkin lymphoma	Case-control study	GnRH: 10;Control: 10	Chemotherapy: ABVD (Adriamycin, Bleomycin, Vinblasin, Dacarbacin), OPPA(Oncovin, Procarbacin, Prednison, Adriamycin) COPP (Cyclophosphamid, Oncovin, Procarbacin,Prednison) BEA-COPP-14 (Bleomycin, Etoposid, Adriamycin, Cyclophosph-amid, Oncovin, Procarbacin, Prednison);GnRH:euprorelin 3.57 mg s.c or goserelin 3.6 mg s.c. monthly before and during chemo-therapy	Resumption of menses,FSH, inhibin B and AMH levels, antral follicle count.	5
Castelo-Branco et al. Hodgkin lymphoma	Case-control study	GnRH: 30;Control: 26	Chemotherapy: Anthracycline, anthracyclineþ taxane, or CMF-based chemotherapy;GnRH:Triptorelin (3.75 mg)/28 days immediately after diagnosis, 1–2 weeks before initiation of chemotherapy, and every 4 weeks thereafter until the end of chemotherapy	Resumption of menses and serial monitoring of FSH and inhibin A and B levels.	6

### GnRH agonist treatment is associated with a lower POF rate

**Figure 2:**
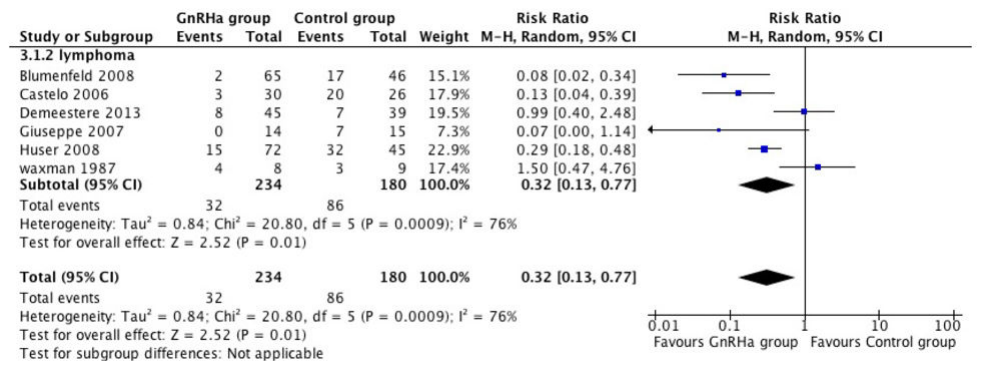
Forest plots showing POF rate of eligible studies comparing GnRH agonists plus chemotherapy with chemotherapy alone.

6 of 7 included articles evaluated POF rate in the last follow-up after cessation of treatment. A substantial heterogeneity was suggested by results of the Q test (P=0.0009) and the I^2^ index (I^2^ Value= 76%). Considering the heterogeneity, we performed the random effect model which showed the incidence of women with POF demonstrated a statistically significant difference in favor of the use of GnRHa (OR=0.32, 95% CI 0.13-0.77). (Figure 2)

### GnRH agonist treatment is not associated with increased spontaneous pregnancy rate

**Figure 3:**
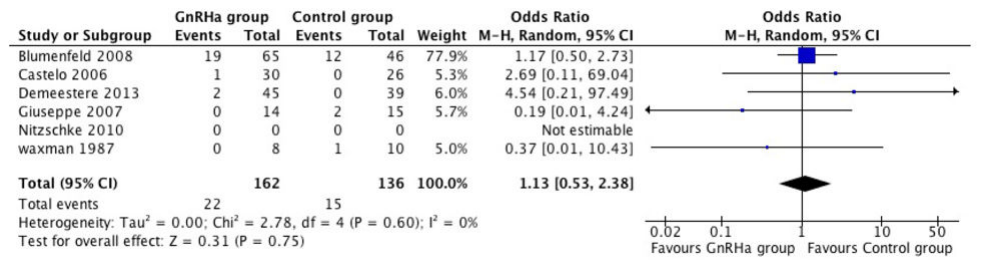
Forest plots showing spontaneous pregnancy rate of eligible studies comparing GnRH agonists plus chemotherapy with chemotherapy alone.

The proportion of women with occurrence of spontaneous pregnancy during the follow-up period after cessation of treatment was evaluated from 6 reports. Since the heterogeneity among the studies was not significant (I^2^ =0%, p=0.60), we used the fixed effect model method. There was no statistically significant difference between treatment and the control groups in the incidence of a spontaneous pregnancy (OR=1.11; 95% CI, 0.55–2.26). (Figure 3)

### GnRH agonist treatment is associated with a lower final FSH level

**Figure 4:**
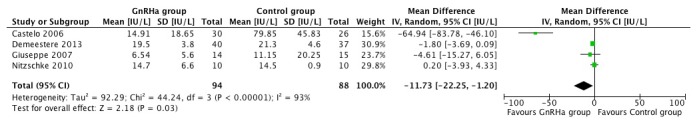
Forest plots showing FSH levels of eligible studies comparing GnRH agonists plus chemotherapy with chemotherapy alone.

4 of 7 included articles evaluated the serum FSH level after cessation of treatment. As the baseline serum FSH data was only available in two studies and there was no significantly difference between the study group and control group, we only used the final FSH level in this meta-analysis. A substantial heterogeneity was suggested by results of the Q test (P=0.000) and the I^2^ index (I^2^ Value= 93%). Since the heterogeneity was significant, we performed the random effect model and the result showed that the final level of FSH in the GnRH group was significantly lower than control group. (MD= -11.73, 95% CI,-22.25- -1.20). (Figure 4)

### GnRH agonist treatment is associated with a lower final anti-Mullerian hormone (AMH) level

**Figure 5:**
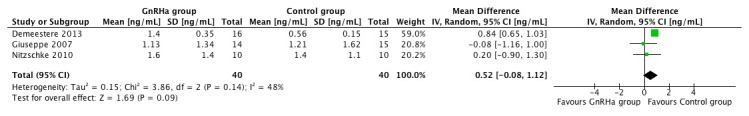
Forest plots showing AMH levels of eligible studies comparing GnRH agonists plus chemotherapy with chemotherapy alone.

3 of 7 included articles evaluated the serum AMH level in the last follow-up after cessation of treatment. As the baseline serum AMH data was not available in the 3 studies, we only used the final AMH level in this meta-analysis. Since the heterogeneity among the three studies was not significant (I^2^ =48%, p=0.14), we used the fixed effect model method. The result showed that the final level of AMH in the GnRH group was significantly higher than control group (MD=0.80; 95% CI, 0.61–0.98). (Figure 5)

## Discussion

This meta-analysis provided clinical evidence that administration of GnRH agonist cotreatment with chemotherapy may be beneficial in preserving future fertility in women treated with chemotherapeutic agents. 

Premature ovarian failure (POF) is characterized by ovarian dysfunction leading to a menopause-like state earlier than 40 years of age. The current clinical assessment criterion for the condition of POF includes primary or secondary amenorrhea lasting more than four consecutive months and serum levels of FSH above 40 IU/l coupled with decreased estrogen levels[15]. However, the studies included in the current meta-analysis used inconsistent definitions of POF (table 1). Some of studies defined POF as no resumption of the menstrual cycle during the follow-up after cessation of chemotherapy and only four studies contained serum FSH level data after chemotherapy. This difference between the studies may have impacted our meta-analysis results. The AMH level has been reported as a suitable marker of ovarian reserve in women treated for lymphoma, reflecting the gonadotoxicity of the drug regimen [16]. Considering the AMH levels, the results suggested that the ovarian reserve was better preserved in the GnRH group. Together, these results suggest that GnRHa may prevent POF in high-risk patients and preserve ovarian reserve of those who recover ovarian function. However, these results must be confirmed by more high quality randomized controlled trials (RCTs). 

It is well-known that a panel of established serum markers of ovarian function or reserve (including AMH, FSH, LH, estradiol, and inhibin-B) is the preferred clinical indicator to detect chemotherapy-related ovarian toxicity. Measurement of AMH coupled with ultrasound-assisted antral follicle counting has been suggested as an effective means of assessing the ovarian reserve. Unfortunately, we were unable to include an analysis of more hormone markers and antral follicle counting to more accurately assess POF or ovarian reserve in the pooled patients from the included studies due to the lack of data.

Other limitations of this meta-analysis include the limited number of included studies, the small number of patients enrolled in each study and the imbalance of the baseline of the subjects. The treatment regimens (of the anti-neoplastic treatment and the GnRH agonist protocol) vary. It is important to note that this difference between the studies may have impacted our meta-analysis results. Moreover, none of the included studies reported how many patients attempted pregnancy in either group, which may contribute to a selection bias. In addition, of the 7 included studies, only 3 studies designed as RCTs provided fair evidence regarding on the unsettled issue. The bulk of the patients included (304 patients) were from non-randomized studies whereas only a small proportion of patients in randomized studies were too small (130 patients included). In some of the studies, the study group is followed prospectively and the control group retrospectively, the time between chemotherapy and evaluation is greater for the control group. The implications are that they are more likely to reach ovarian failure at the time of evaluation.

The lack of RCTs may be a result of the difficulty of enrolling young patients, particularly hematologic patients. Other randomized studies that enrolled patients with breast cancer have been reported, with contradictory results concerning the efficacy of GnRHa in preventing chemotherapy-induced POF. Some studies reported similar rates of menstruation recovery between patients treated with GnRH during chemotherapy and patients treated with chemotherapy alone [17,18]. In contrast, other studies found significant higher rates of menstruation recovery in the GnRH groups[6,19]. Based on the previously studies, two well-designed meta-analyses suggested a potential benefit of GnRHa cotreatment with chemotherapy in premenopausal women[20,21]. In each of these meta-analyses, however, there was substantial heterogeneity in the types of disease, including hematologic diseases (lymphoma and leukemia) and breast cancer. Another meta-analysis provided evidence that the administration of GnRHa during chemotherapy treatment in premenopausal women appears to protect against chemotherapy-related POF in breast cancer patients[22]. Since the median age of the cohorts of patients with breast cancer was more than 36 years, the younger patients with lymphoma represent a group that is potentially more concerned with fertility preservation. To the best of our knowledge, this is the first meta-analysis concentrated on the effect of the preservation of ovarian function by administering GnRHa in young patients with lymphoma undergoing chemotherapy. 

In addition to the potential protective effects of GnRHa on ovarian function, this treatment may reduce the occurrence of hypermenorrhea during chemotherapy. One included study showed that vaginal bleeding occurred less frequently in the GnRHa group than in the control group during chemotherapy[7]. On the other hand, GnRH agonists have side effects such as hot flashes and decreased bone density. One included study compared the change of bone mineral density in GnRH group and control group, and the result showed there is no significant difference between the two groups[9]. It is accepted that the loss of bone mineral density in this situation may be reduced significantly by using low doses of hormone replacement therapy[23]. Thus, if GnRHa is considered the treatment of choice, then hormone replacement therapy should be used in combination.

In conclusion, the results of this meta-analysis suggest that GnRHa may be effective in protecting ovarian function during chemotherapy in lymphoma patients. However, more high quality, well designed, randomized, controlled, multicenter trials are needed to confirm these results.

## Supporting Information

Checklist S1 **PRISMA Checklist.**
(DOC)
